# Effects of tocilizumab and dexamethasone on the downregulation of proinflammatory cytokines and upregulation of antioxidants in the lungs in oleic acid-induced ARDS

**DOI:** 10.1186/s12931-022-02172-w

**Published:** 2022-09-17

**Authors:** Funda Terzi, Beste Demirci, İrfan Çınar, Mohammad Alhilal, Huseyin Serkan Erol

**Affiliations:** 1 Department of Pathology, Faculty of Veterinary Medicine, Kastamonu University, Kuzeykent Campus, 37150 Kastamonu, Turkey; 2 Department of Anatomy, Faculty of Veterinary Medicine, Kastamonu University, 37150 Kastamonu, Turkey; 3 Department of Medicinal Pharmacology, Faculty of Medicine, Kastamonu University, 37150 Kastamonu, Turkey; 4 Department of Nursing, Faculty of Health Sciences, Mardin Artuklu University, 47200 Mardin, Turkey; 5 Department of Biochemistry, Faculty of Veterinary Medicine, Kastamonu University, 37150 Kastamonu, Turkey

**Keywords:** ARDS, Tocilizumab, Dexamethasone, Oleic acid, Rat

## Abstract

**Background:**

Acute respiratory distress syndrome (ARDS) is a life-threatening disease caused by the induction of inflammatory cytokines and chemokines in the lungs. There is a dearth of drug applications that can be used to prevent cytokine storms in ARDS treatment. This study was designed to investigate the effects of tocilizumab and dexamethasone on oxidative stress, antioxidant parameters, and cytokine storms in acute lung injury caused by oleic acid in rats.

**Methods:**

Adult male rats were divided into five groups: the CN (healthy rats, n = 6), OA (oleic acid administration, n = 6), OA + TCZ-2 (oleic acid and tocilizumab at 2 mg/kg, n = 6), OA + TCZ-4 (oleic acid and tocilizumab at 4 mg/kg, n = 6), and OA + DEX-10 (oleic acid and dexamethasone at 10 mg/kg, n = 6) groups. All animals were euthanized after treatment for histopathological, immunohistochemical, biochemical, PCR, and SEM analyses.

**Results:**

Expressions of TNF-α, IL-1β, IL-6, and IL-8 cytokines in rats with acute lung injury induced by oleic acid were downregulated in the TCZ and DEX groups compared to the OA group (P < 0.05). The MDA level in lung tissues was statistically lower in the OA + TCZ-4 group compared to the OA group. It was further determined that SOD, GSH, and CAT levels were decreased in the OA group and increased in the TCZ and DEX groups (P < 0.05). Histopathological findings such as thickening of the alveoli, hyperemia, and peribronchial cell infiltration were found to be similar when lung tissues of the TCZ and DEX groups were compared to the control group. With SEM imaging of the lung tissues, it was found that the alveolar lining layer had become indistinct in the OA, OA + TCZ-2, and OA + TCZ-4 groups.

**Conclusions:**

In this model of acute lung injury caused by oleic acid, tocilizumab and dexamethasone were effective in preventing cytokine storms by downregulating the expression of proinflammatory cytokines including TNF-α, IL-1β, IL-6, and IL-8. Against the downregulation of antioxidant parameters such as SOD and GSH in the lung tissues caused by oleic acid, tocilizumab and dexamethasone upregulated them and showed protective effects against cell damage.

## Background

Acute lung injury (ALI), also known as acute respiratory distress syndrome (ARDS) in more severe cases, is a fatal inflammatory process often induced by sepsis, trauma, pneumonia, burns, multiple organ transplants, cardiopulmonary operations, and pancreatitis [1, 2]. Pathological changes such as the deterioration of pulmonary vascular permeability, pulmonary edema, hyaline membrane formation, microatelectasis of alveolar epithelial cells, microthrombosis, and microcirculation disorders are seen in ARDS [3–5]. Oxidative stress and inflammatory response are two important factors in ARDS [6].

In ALI/ARDS, the inflammatory response is initiated by a complex network of cytokines and other proinflammatory molecules produced by various types of cells in the lungs, including inflammatory cells with the recruitment of blood leukocytes and activation of tissue macrophages [6]. The early-response proinflammatory cytokines are tumor necrosis factor α (TNF-α) and interleukin (IL)-1β, which are produced by macrophages, neutrophils, and other cell types [7, 8]. TNF-α and IL-1β act locally on other cells, including monocytes/macrophages, endothelial cells, fibroblasts, and epithelial cells, and they stimulate the production of other cytokines such as IL-2, IL-4, IL-6, and IL-8 [6]. In patients with ARDS, inflammatory cytokines such as IL-1β, TNF-α, IL-6, and IL-8 are elevated in the bronchoalveolar lavage fluid and plasma [9, 10]. IL-6 is believed to play a crucial role in the development of cytokine storms, contributing to the occurrence of ARDS and causing interstitial pneumonia in patients with severe COVID-19 [11].

Reactive oxygen species (ROS) are produced in large amounts by damaged endothelial/epithelial cells as well as leukocytes, leading to the aggravation of ARDS through lipid peroxidation, which can alter both the structure and function of pulmonary capillaries [12]. Antioxidant enzymes such as superoxide dismutase (SOD), catalase (CAT), glutathione peroxidase (GPx), and glutathione (GSH) are naturally present in the lungs, but the intrapulmonary levels of these enzymes can be greatly elevated when the lungs are faced with an oxidant burst that disrupts the redox balance, such as in cases of hyperoxia [13]. SOD catalyzes superoxide anion radicals to hydrogen peroxide (H_2_O_2_) and oxygen, while CAT converts H_2_O_2_ to water and oxygen [14]. GSH scavenges various radicals and also acts as a direct antioxidant by participating in glutathione peroxidase reactions [15].

Tocilizumab (TCZ) is a recombinant humanized monoclonal antibody that also binds to its soluble forms [16]. The mechanism of TCZ blocks receptor complex signaling to inflammatory mediators responsible for B-cell and T-cell activation and inhibits cytokine storms [17]. This drug is used in the treatment of certain autoimmune disorders such as rheumatoid arthritis, juvenile idiopathic arthritis, and Castleman disease [18]. In more recent years, TCZ has been widely utilized in the treatment of COVID-19, which may cause acute respiratory syndrome [19].

In the treatment of ALI/ARDS, glucocorticoids have been used for many years as they reduce inflammation and fibrosis through the inhibition of various cytokines, including IL-1, IL-3, IL-5, IL-6, IL-8, and TNF-α [20, 21]. Dexamethasone (DEX), one of these steroids, reduces the production of inflammatory cytokines and pulmonary edema while also alleviating alveolar epithelial and endothelial cell damage [22]. In this study, it was aimed to investigate the efficacy of TCZ and DEX on oxidative stress, antioxidant parameters, and cytokine storms in a rat model of ALI caused by oleic acid.

## Materials and methods

### Animals

A total of 30 adult male Wistar albino rats aged 12–13 weeks and weighing 200–220 g were purchased from the Bolu Abant İzzet Baysal University Medical Experimental Research and Application Center. Rats were kept at temperatures ranging between 19 °C and 22 °C with a standard 12-h light/dark cycle. The experimental animal model of ALI/ARDS was achieved with oleic acid (OA). All the practices on rats were carried out with reference to European Union Directive and were approved by the local ethics committee of Kastamonu University (approval no: 2020/31).

### Experimental procedures

Adult male rats were randomly selected and divided into five groups: the CN (healthy rats, n = 6), OA (oleic acid administration, n = 6), OA + TCZ-2 (oleic acid and tocilizumab at 2 mg/kg, n = 6), OA + TCZ-4 (oleic acid and tocilizumab at 4 mg/kg, n = 6), and OA + DEX-10 (oleic acid and dexamethasone at 10 mg/kg, n = 6) groups. Oleic acid (50 µL) was dissolved in 250 µL of 1% bovine serum albumin and administered via the tail vein in all groups except the CN group. DEX (Dekort Ampul 8 mg/2 mL, Deva Ilac, Turkey) and TCZ (Actemra, Roche, Germany) were administered intraperitoneally twice 6 h after OA injection at an interval of 12 h using an insulin injector. The rats were anesthetized by administering intraperitoneal ketamine hydrochloride (60 mg/kg b.w) and xylazine hydrochloride (5 mg/kg b.w) [23]. Subsequently, cervical dislocation was applied to the animals. Systemic autopsies were performed and lung tissues were taken for biochemical and pathological analysis. Lung tissues were stored at − 20 °C for biochemical analysis and were kept in NBF for pathological analysis.

### Histopathological analysis

The tissues were cut and moved to tissue cassettes. Routine pathology follow-up was performed after the cassettes were cleaned under running water and a paraffin block was applied. Hematoxylin and eosin staining was performed for pieces of paraffin blocks 5 µm in thickness, which were cut with a microtome and mounted on adhesive slides with coverslips. Sections were subsequently examined under a light microscope. Histopathological changes were scored semiquantitatively as follows: − (0): absent; + (1): mild; ++ (2): moderate; +++ (3): severe [24].

### Immunohistochemical analysis

Immunohistochemical staining was performed according to the manufacturer’s procedure with the Mouse and Rabbit Specific HRP/DAB IHC Detection Kit-Micro-polymer (Cat No. ab236466; Abcam, UK). Proteinase K (Cat No. ab64220; Abcam, UK) was used for antigen retrieval. Sections taken from paraffin blocks with adhesive were submerged in 3% H_2_O_2_ peroxidase block solution and then the protein block solution was poured out. The sections were exposed to 1:100 anti-TNF-α antibody (Cat No. ab6671; Abcam, USA), anti-IL-6 antibody (Cat No. ab6672; Abcam, USA), and anti-IL-8 antibody (Cat No. ab34100; Abcam, USA) and were left at room temperature for 1 h. Subsequently, Mouse Identification Reagent (Complementary) solution was added to the slides and they were exposed to goat anti-rabbit HRP-conjugate. Slides were stained with DAB (3,3′-diaminobenzidine tetrahydrochloride). After counterstaining with Mayer’s hematoxylin, slides were sealed with coverslips and evaluated under a light microscope (Leica DM 400B, Leica, Germany). Negative control slides were also stained according to the same procedure and PBS was used instead of primary antibodies. Immunohistochemical staining was scored semiquantitatively as revealing low (+), moderate (++), or high (+++) expression.

### Scanning electron microscopy (SEM) analysis

Tissue samples of 5 × 5 × 5 mm were taken from the peripheral ends of two rat lungs and washed in PBS. These tissues were then fixed in 2.5% glutaraldehyde and kept in 1% OsO_4_, and then they were passed through a graded acetone series. All tissue fixation steps were performed under a fume hood. Tissues were dried in a critical point dryer (E3100, Quorum Technologies, UK) and then coated with gold palladium (Au–Pd; 108 Auto Sputter Coater, Cressington, UK). They were examined and visualized under high vacuum at 5.00 kV with an ETD detector and scanning electron microscope (FEI Quanta FEG-250, Thermo Fisher Scientific, USA). SEM analysis was performed in the Imaging Laboratory of Kastamonu University’s Central Research Laboratory.

### Biochemical analyses

#### Preparation of tissue homogenates

Tissues from petri dishes were pulverized with liquid nitrogen in a porcelain mortar. They were then weighed to 25 mg in sterile Eppendorf tubes with the addition of homogenate buffers suitable for the enzymes being evaluated (LPO: 10% KCl; SOD: 50 mM KH_2_PO_4_, 10 mM EDTA; GSH: 50 mM Tris–HCl; CAT: 50 mM KH_2_PO_4_, pH 7). Tissues in the buffers were homogenized for 1 min at a frequency of 35 Hz using a tissue homogenizer (TissueLyser II, QIAGEN, Germany) with a 5-mm steel ball. Subsequently, the homogenates were centrifuged in a refrigerated centrifuge (Universal 320 R, Hettich GmbH & Co. KG, Germany) at 4 °C and 4000 rpm for 30 min for LPO and GSH, 6000 rpm for 1 h for SOD, and 8500 rpm for 1 h for CAT. The resulting supernatants were then used in accordance with the relevant measurement methods.

#### Determination of lipid peroxidation (LPO) levels

For the determination of lung tissue LPO levels, the method based on the reaction between thiobarbituric acid and malondialdehyde described by Ohkawa et al. [25] was used. In the calculation of tissue LPO level, absorbances read at 532 nm by spectrophotometer (Bio-Tek EPOCH, Bio-Tek, USA) were calculated using a standard graph created with 1,1,3,3-tetramethoxypropane and the results were expressed as nmol MDA/g tissue.

#### Determination of superoxide dismutase (SOD) enzyme activity

For the determination of lung tissue SOD activities, a method based on the detection of formazan dye reduction by superoxide radicals with xanthine oxidase activity was used [26]. To determine the activity, absorbances read at 560 nm were calculated with the equation specified in the cited reference and were expressed as U/mg tissue.

#### Determination of catalase (CAT) enzyme activity

CAT activities in lung tissues were determined using a method that utilizes the conversion of H_2_O_2_ to water by an enzymatic reaction [27]. After the samples were read kinetically at 240 nm, they were calculated with the formula specified in the cited reference and expressed as µmol/min/mg tissue.

#### Determination of glutathione (GSH) levels

GSH levels of lung tissues were determined according to a previously described method [28]. GSH levels of the tissues were determined at 412 nm and expressed as nmol/mg lung tissue.

### Molecular analysis of gene expressions

#### RNA isolation and cDNA synthesis

Lung tissues were stabilized in RNA Stabilization Reagent (RNAlater, QIAGEN, Germany), then homogenized with the TissueLyser II device (QIAGEN). Total RNA was isolated from homogenized lung tissues following the instructions provided for the RNeasy Mini Kit (QIAGEN). cDNA was synthesized by reverse transcription of complementary DNA from isolated RNA samples according to the instructions of the High-Capacity cDNA Reverse Transcription Kit (Applied Biosystems, USA), as previously described [29]. The sequences of PCR primer pairs used for each gene are shown in Table 1.

**Table 1:** Primers used in real-time PCR experiments

Gene name	Primer sequence	Accession number
TNF-α	F: 5′-CCAGGAGAAAGTCAGCCTCCT-3′ F: 5′-TCATACCAGGGCTTGAGCTCA-3′	X66539
IL-1β	F: 5′-CACCTCTCAAGCAGAGCACAG-3′ R: 5′-GGGTTCCATGGTGAAGTCAAC-3′	NM_031512
IL-6	F: 5′-TCCTACCCCAACTTCCAATGCTC-3′ R: 5′-TTGGATGGTCTTGGTCCTTAGCC-3′	NM_012589
β-actin	F: 5′-AGGCCGGCTTCGCGGGCGAC-3′ R: 5′-CTCGGGAGCCACACGCAGCTC-3′	NM_031144.3

#### Relative analysis of gene expressions (real-time quantitative PCR)

Expression analysis of TNF-α, IL-1β, and IL-6 as target genes was conducted with the StepOnePlus Real-Time PCR System (Applied Biosystems, USA) for each cDNA sample synthesized from rat lung RNA. The expression analysis of β-actin was used as an endogenous reference gene. Quantitative real-time PCR was conducted with One-Step TaqMan Gene Expression Assays Probe-based technology (Primer Design Ltd., UK), as previously described [30]. The obtained data were expressed as fold changes in expression using the 2^−ΔΔCt^ method and compared to the values of the healthy control group [31].

### Statistical analysis

IBM SPSS Statistics 25.0 (IBM Corp., USA) was used to compare the histopathology results. All values are presented as mean ± standard deviation. Biochemical analyses and mRNA expression levels were statistically analyzed with one-way analysis of variance (ANOVA) among study groups. Significant differences were determined after the data were analyzed by Tukey’s multiple range test. Histopathological and immunohistochemical scores were analyzed using Kruskal–Wallis and Mann–Whitney U tests. Values of P < 0.05 were considered statistically significant.

## Results

### Histopathological results

**Fig. 1:**
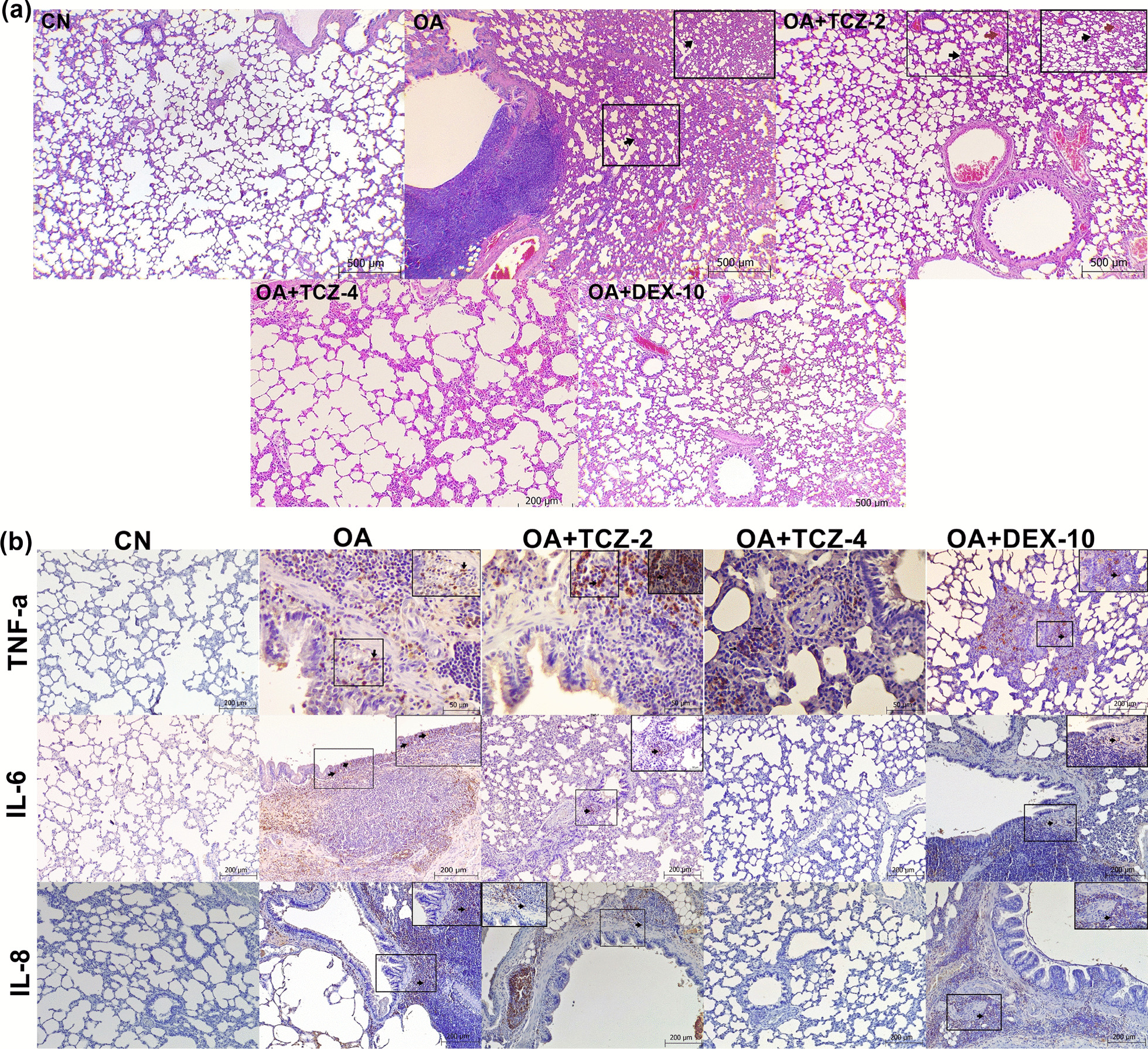
**a** Pathological changes such as peribronchial hyperplasia in lymphoid tissues, thickening of the interalveolar septum (black arrows), and hyperemia (red arrows) of the interalveolar septum were detected in the lungs by H&E staining. Bars: 200 and 500 µm. **b** Expressions of TNF-α, IL-6, and IL-8 were evaluated by immunohistochemistry. Bars: 50 and 200 µm

Histopathological findings in the lungs of the control and experimental groups are summarized in Table 2. In lung tissue from OA group rats was detected extensive alveolar damage including increased vascular permeability, intraalveolar hyaline membrane formation and intense interstitial intraalveolar infiltration by mononuclear cells. Thickening of the interalveolar septum in the lungs was statistically more severe in the OA group than in the other experimental groups (P < 0.05). In addition, there was no statistically significant difference between the thickening of the interalveolar septum seen in the CN, OA + TCZ-4, and OA + DEX-10 groups (P > 0.05) (Fig. 1a). Peribronchial cell infiltration was found to be statistically more severe in the OA group than in the TCZ and DEX groups (P < 0.05). The vessels in the lung interalveolar septum were statistically more severely affected in the OA group (P < 0.05), while there was no statistically significant difference between the CN group and OA + TCZ (2 and 4) and OA + DEX-10 groups (P > 0.05).

### Immunohistochemical results

The immunohistochemical method was used to determine the expressions of TNF-α, IL-6, and IL-8 cytokines in the lung tissues of rats from the control and experimental groups. The results are summarized in Table 2. TNF-α expression was statistically significantly increased in the OA group compared to the other groups (P < 0.05) (Fig. 1b). There was no significant difference in TNF-α expression between the OA + TCZ-2, OA + TCZ-4 and OA + DEX-10 groups (P > 0.05). IL-6 expression was statistically significantly increased in the OA group compared to the OA + TCZ-4 and OA + DEX-10 groups (P < 0.05) (Fig. 1b). IL-6 expression was detected in the OA group and moderate staining was observed in the OA + TCZ-2 group. IL-8 expression was significantly increased in the OA group compared to the other experimental groups (P < 0.05). Moderate IL-8 staining was observed in the OA + TCZ-2 and OA + DEX-10 groups, while it was negative in the OA-TCZ-4 and CN groups.

**Table 2:** Immunohistochemical and histopathological findings for the effects of on oleic acid-induced acute lung injury

		CN	OA	OA + TCZ-2	OA + TCZ-4	OA + DEX-10	P-value
Histopathological	Thickening in the interalveolar septum	0.60 ± 0.54^b^	2.40 ± 0.54^a^	1.40 ± 0.54^ab^	1.00 ± 0.70^b^	0.80 ± 0.44^b^	0.006
	Peribronchial cell infiltration	0.60 ± 0.54^b^	2.88 ± 0.40^a^	1.20 ± 0.44^b^	1.00 ± 0.70^b^	1.00 ± 0.70^b^	0.006
	Hyperemia in vessels	1.20 ± 0.44^b^	2.60 ± 0.54^a^	1.40 ± 0.54^b^	1.40 ± 0.89^b^	1.40 ± 0.54^b^	0.039
Immunohistochemical	TNF-a	0.50 ± 0.57^b^	2.75 ± 0.50^a^	1.75 ± 0.95^ab^	2.5 ± 1.00^ab^	1.5 ± 1.00^ab^	0.034
	IL-6	1.00 ± 1.15^b^	2.50 ± 0.57^a^	1.25 ± 0.5^ab^	0.50 ± 0.57^b^	0.75 ± 0.50^b^	0.41
	IL-8	0.75 ± 0.50^b^	2.5 ± 0.57^a^	2.00 ± 0.81^ab^	0.25 ± 0.50^b^	0.75 ± 0.95^b^	0.13

^a, b^P < 0.05. All values are given as mean ± standard deviation

### SEM results

**Fig. 2:**
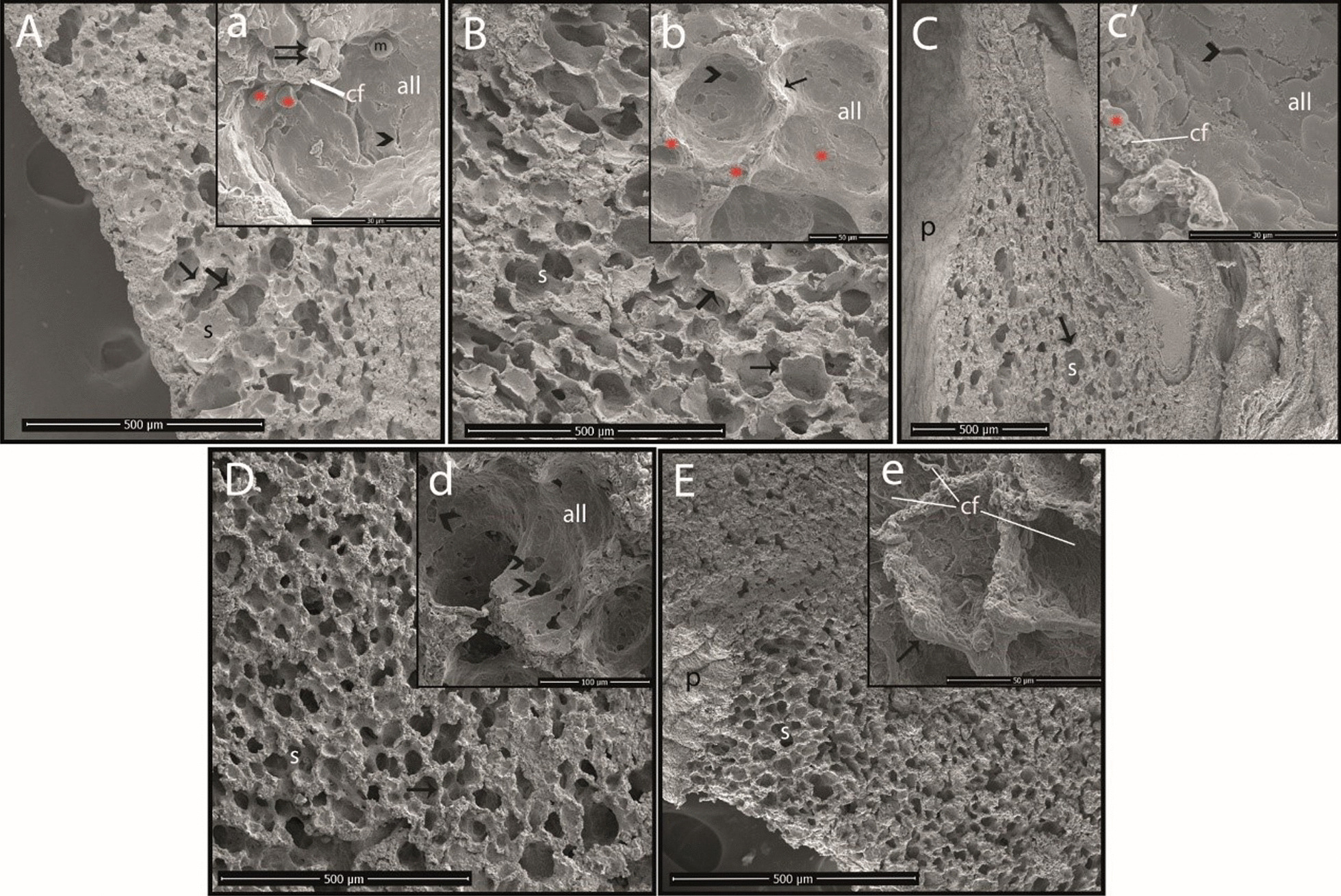
SEM images of the rat lungs. **A** Cross-sectional area from the control group (bar: 500 µm), **a** alveolar surface of the control group (bar: 30 µm). **B** Cross-sectional area from the OA group (bar: 500 µm), **b** alveolar surface of the OA group (bar: 50 µm). **C** Cross-sectional area from the OA + DEX-10 group (bar: 500 µm), **c**′ alveolar surface of the OA + DEX-10 group (bar: 30 µm). **D** Cross-sectional area from the OA + TCZ-2 group (bar: 500 µm), **d** alveolar surface of the OA + TCZ-2 group (bar: 100 µm). **E** Cross-sectional area from the OA + TCZ-4 group (bar: 500 µm), **e** alveolar surface of the OA + TCZ-4 group (bar: 50 µm). **˃**: Kohn pore; *: erythrocyte; arrow: interalveolar septum; double arrow: alveolar capillary vessel; p: pleura; s: alveolar sac; m: alveolar macrophage; all: alveolar lining layer; cf: collagen fibrils

All lung tissues were carefully examined by SEM. In this process, a thin layer of alveolar lining was detected inside the alveolar sacs (Fig. 2A and D). In addition, alveolar macrophages and erythrocytes were prominent (Fig. 2A and C). Collagen fibrils and alveolar capillary vessels were observed in the interalveolar septum. Alveolar capillary vessels were found to be full in the OA group (Fig. 2B). It was further observed that the cells forming the alveolar lining layer had become indistinct in the OA, OA + TCZ-2, and OA + TCZ-4 groups compared to the CN and OA + DEX-10 groups (Fig. 2). In the OA + TCZ-4 group, the cells forming the alveolar lining layer had completely deteriorated, the collagen fibrils under this layer were exposed, and the vascular structures had deteriorated (Fig. 2E). In addition, collateral channels known as Kohn pores were observed as rather large and emphysematous structures in the OA + TCZ-2 group (Fig. 2D).

### Biochemical results

**Fig. 3:**
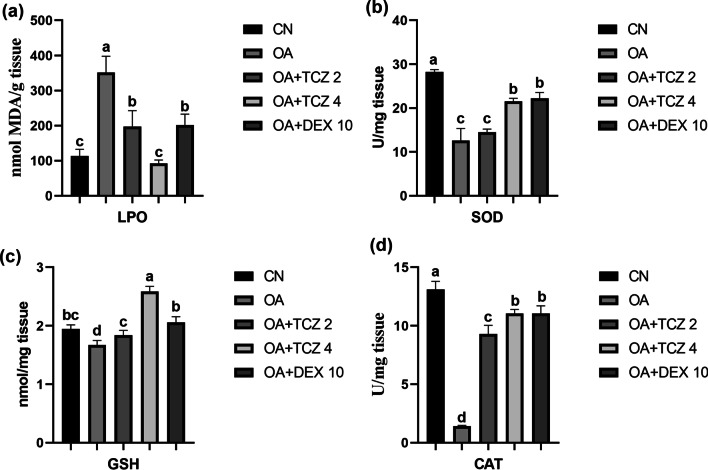
**a** In lung tissues, LPO levels were significantly increased in the OA group (P < 0.05) and were significantly decreased in the DEX and TCZ groups (P < 0.05). **b** SOD activity was significantly decreased in the OA and OA + TCZ-2 groups (P < 0.05), while it was significantly increased in the OA + TCZ-4 and OA + DEX-10 groups compared to the OA group (P < 0.05). **c** In the OA + TCZ-4 group, GSH levels were significantly increased compared to the OA and other experimental groups (P < 0.05). **d** CAT activity was significantly decreased in the OA group (P < 0.05), while it was significantly increased in all other experimental groups compared to the OA group (P < 0.05). Different letters (a, b, c, d) above bars indicate statistical differences between control and experimental groups according to Tukey’s multiple range test (P < 0.05)

In the lung tissues, LPO levels were increased statistically in the OA group compared to the control group (P < 0.05). However, the LPO levels were not statistically significantly different between the OA + TCZ-2 and OA + DEX-10 groups or the CN and OA + TCZ-4 groups (P > 0.05) (Fig. 3a). SOD activity was significantly lower in the OA and OA + TCZ-2 groups compared to the OA + TCZ-4 and OA + DEX-10 groups (P < 0.05). In addition, SOD activity was not statistically significantly different in the OA + TCZ-4 and OA + DEX-10 groups (P > 0.05) (Fig. 3b). GSH levels increased significantly in the OA + TCZ-4 group compared to the OA group (P < 0.05), while the GSH levels of the CN, OA + TCZ-2, and OA + DEX-10 groups were statistically similar (P < 0.05) (Fig. 3c). CAT activity was significantly reduced in the OA group (P < 0.05). There was no statistically significant difference between CAT activities in the OA + TCZ-4 and OA + DEX-10 groups (P > 0.05) (Fig. 3d).

### Molecular results

**Fig. 4:**
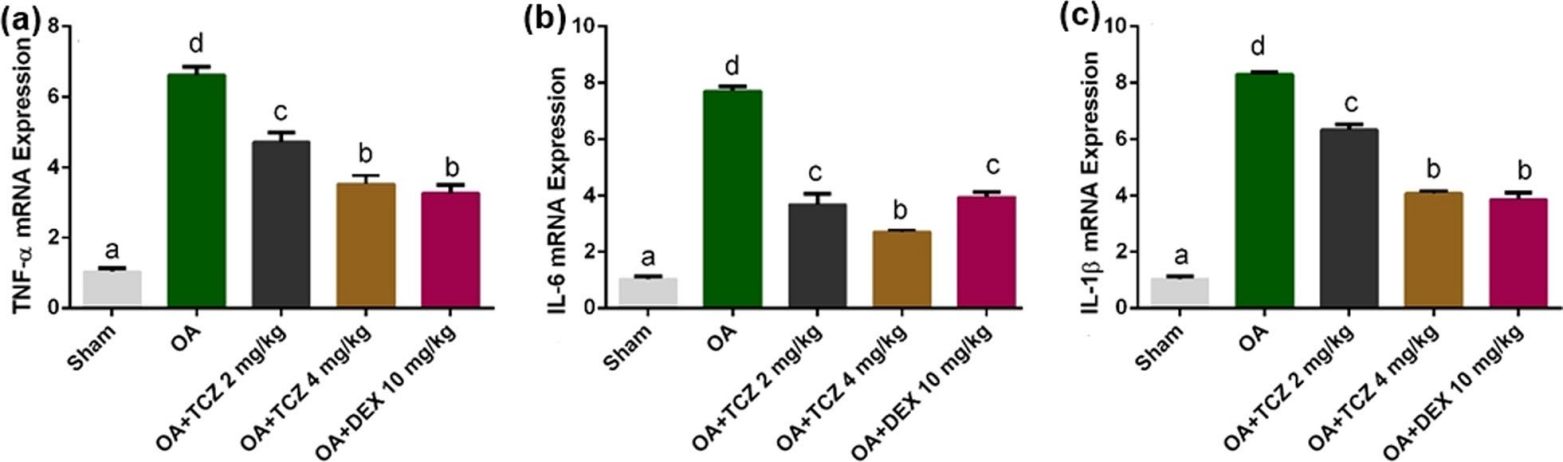
**a**–**c** Effects of TCZ and DEX on OA-induced changes in lung tissues according to relative mRNA expressions of TNF-α, IL-1β, and IL-6. Each bar with a vertical line represents the mean fold change ± SEM of 6 rats compared to the control group. OA significantly increased TNF-α, IL-1β, and IL-6 levels in lung tissues compared to the control group (P < 0.05). All experimental treatments markedly decreased the TNF-α, IL-1β, and IL-6 levels in the lung tissues compared to the OA group (P < 0.05). Different letters (*a*, *b*, *c*, *d*) above bars indicate statistical differences between groups according to Duncan’s multiple range test (P < 0.05). There is no statistically significant difference between groups marked with the same letters in a graph (P > 0.05)

To evaluate whether TCZ (2 and 4 mg/kg) or DEX (10 mg/kg) attenuated OA-induced ARDS, the expression levels of TNF-α, IL-1β, and IL-6 mRNA in the lung tissues of the rats were analyzed using real-time PCR (Fig. 4a–c). It was observed that OA administration caused significant increases in the expressions of TNF-α, IL-1β, and IL-6 mRNA levels in the lungs (P < 0.05). On the other hand, treatment with TCZ at 4 mg/kg and DEX at 10 mg/kg led to significant decreases in TNF-α, IL-1β, and IL-6 mRNA expression levels when compared to the OA group (P < 0.05). There was no statistically significant difference in TNF-α and IL1β expression in the OA + TCZ-4 and OA + DEX-10 groups (P > 0.05). In addition, there was no statistically significant difference in IL-6 expression between the OA + TCZ-2 and OA + DEX-10 groups (P > 0.05).

## Discussion

ALI and its more severe form, ARDS, occur due to various clinical conditions such as trauma, aspiration, sepsis, endotoxemia, and pneumonia [32]. The pathophysiology of ARDS is quite complex [33]. In ARDS, proinflammatory cytokines and interconnected inflammatory cascades are important for the inflammatory response [34]. As a result of this inflammatory response, the permeability of alveolar capillaries increases, microthrombi form, and hypoxic pulmonary vasoconstriction is impaired. Endothelial and epithelial damage occurs with the imbalance of the ventilation-perfusion relationship, leading to alveolar edema, decreased lung compliance, and, ultimately, refractory hypoxemia [6]. In the treatment of the disease, glucocorticoids, surfactants, inhaled nitric oxide, antioxidants, protease inhibitors, and various other anti-inflammatories are used [35]. OA-induced lung injury, a well-defined laboratory model of ALI, is the most commonly preferred model for the evaluation of potential therapeutic agents [36, 37]. In the present study, it was aimed to investigate the effects of TCZ and DEX on cytokine storms, oxidative stress, and antioxidants in rats with ALI caused by OA.

In our experimental study, we determined that OA has an important role in cytokine storms in lung tissues. Some proinflammatory cytokines have prognostic significance in the pathogenesis of ARDS [9]. In cases of COVID-19 characterized by ARDS, IL-6 is believed to play a role in the development of cytokine storms [38]. IL-6 inhibitors are widely available for use in the treatment of COVID-19 as monoclonal antibodies targeting IL-6 (siltuximab) or IL-6R (tocilizumab and sarilumab). TCZ is a neutralizing antibody against IL-6 and IL-6R and blocks both classical and signal transduction pathways [36], and it is currently the most preferred IL-6 inhibitor in the treatment of patients with COVID-19/ARDS. In a study conducted to determine the appropriate dose of tocilizumab, it was found that TCZ at 2 mg/kg was effective in reducing the severity of severe acute pancreatitis and ALI [18]. The same study also found a diminishing benefit in attenuating morphological changes in the lungs when the dose of TCZ was increased by more than 4 mg/kg. In an LPS-induced ALI model, it was found that a TCZ dose of 10 mg/kg significantly reduced the release of proinflammatory cytokines [39]. However, tocilizumab at a dose of 8 mg/kg may be of maximum benefit in patients with rheumatoid arthritis [40], streptozotocin-induced diabetic nephropathy [41], and cerulein-induced experimental acute pancreatitis [42]. In our experimental study, we determined via histopathological and molecular methods that TCZ at a dose of 4 mg/kg was more effective in reducing the severity of ARDS.

IL-6 levels are positively correlated with the production of proinflammatory cytokines and chemokines [43]. TNF-α and IL-1β are mostly produced by activated macrophages and act via specific cell membrane-bound receptors. These cytokines stimulate the expression of IL-6 and IL-8 in other cells, including monocytes/macrophages, endothelial cells, fibroblasts, and epithelial cells [6]. In our study, we found that OA increased the expressions of TNF-α, IL-1β, IL-6, and IL-8 in lung tissues, and TCZ 4 mg/kg decreased these proinflammatory cytokines expressions. The findings of our study also confirmed that IL-6 is a cytokine with prognostic significance in cases of ARDS and has a synergistic effect with other proinflammatory cytokines.

Glucocorticoids have long been used to treat ARDS [44]. DEX, a synthetic corticosteroid, acts as a broad-spectrum immunosuppressive and has greater activity and longer duration of action than cortisone [45]. The drug reduces the severity of ARDS and improves patient prognosis with its potential to reduce inflammation in the lungs [46]. In a previous study [44], it was determined that serum TNF-α, IL-6, and VEGF levels of rats treated with OA and DEX were significantly lower than those of rats receiving only OA. In our experimental study, we determined that DEX (10 mg/kg) and TCZ (4 mg/kg) significantly decreased the expression of TNF-α, IL-1β, IL-6, and IL-8. DEX is thought to be useful in the hyperinflammation or cytokine storms associated with COVID-19/ARDS [47]. However, since DEX has serious side effects including hyperglycemia, fatty liver, insulin resistance, and type II diabetes, TCZ is often recommended to be used in the treatment of ARDS patients.

Similar to previous studies [24, 48], MDA levels were found to be high in lung tissues in the present work and OA caused damage to lung cells. ROS such as superoxide anion radical, H_2_O_2_, and hydroxyl radical cause the oxidative breakdown of the polyunsaturated fatty acids of cell membranes, a process known as lipid peroxidation [49], and MDA is formed in the end. It was determined here that the MDA levels in lung tissues were significantly lower in rats administered TCZ at 4 mg/kg compared to DEX at 10 mg/kg. TCZ is thought to be more effective than DEX in reducing oxidative stress and free radical reactions in response to OA treatment.

In our study, we determined that OA significantly decreased antioxidant parameters in lung tissues while TCZ and DEX reversed that damage. H_2_O_2_ has an important role in the pathogenesis of ALI/ARDS [50]. SOD catalyzes superoxide radicals in a dismutation reaction that produces H_2_O_2_, while CAT and GSH enable the conversion of H_2_O_2_ to water and oxygen [51]. GSH is an intracellular thiol found at high levels in all tissues and various body fluids, particularly lung tissues and bronchoalveolar lavage fluid [52]. GSH is oxidized to GSSG via the enzyme glutathione peroxidase (GPx), while GSSG is reduced to GSH via glutathione reductase (GR). GSH acts as an antioxidant by repairing cellular damage [53] and helps relieve inflammation [54]. In experimental studies, it has been determined that OA reduces SOD, GSH, and CAT activities in lung tissues [24, 32, 55, 56]. In the present work, TCZ and DEX showed antioxidant effects against oxidative stress caused by OA by increasing SOD, GSH, and CAT activities.

OA induces acute diffuse lung injury in rats similar to ALI and ARDS in humans [32]. It causes focal bleeding and vascular congestion in lung tissues, diffuse interstitial and alveolar edema, and interstitial and alveolar infiltration of leukocytes [37]. Histopathological findings such as alveolar thickening, hyperemia, and peribronchial cell infiltration in the lung tissues of rats administered OA were severe in this study, while the lung tissues of rats in the TCZ and DEX groups were histopathologically similar to those of the control group. In addition, it was seen in SEM imaging that blood vessels in the lungs of rats of the OA group were full and the cells forming the alveolar lining layer had become indistinct in the OA, OA + TCZ-2, and OA + TCZ-4 groups. In the OA + TCZ-4 group, it was noted that the cells forming the alveolar lining layer were completely disrupted, the collagen fibrils under this layer were exposed, and the vascular structures had deteriorated. We believe that the findings of this study will contribute to future studies on SEM imaging of the lungs.

The strengths of this study include its investigation of the effects of tocilizumab and dexamethasone, which are widely used in the treatment of COVID-19 patients today, in preventing cytokine storms in an animal model of oleic acid-induced ARDS by molecular methods. However, our approach has some limitations. First, we could not perform respiratory function tests for the ARDS and treatment groups. We could not perform chest X-rays, CT scans, or blood gas (PaO_2_, PaCO_2_, and pH) measurements to further evaluate the development of oleic acid-induced ARDS. Our ARDS model in rats was evaluated using lung tissue histopathological data. However, previous reports have confirmed that histopathological data are sufficient in confirming ARDS models [24, 57]. Secondly, we determined changes in the expression of TNF-α, a proinflammatory cytokine, in the lung tissues of oleic-induced ARDS cases and treatment groups. TNF-α also plays a role in the mechanism of apoptosis, and in our study, we could not examine markers in apoptosis pathways at the cellular level. Finally, due to the lack of appropriate doses at which TCZ and DEX could be used together, a group in which the two treatment options were used together was not formed in our study. However, in our study, we did not detect a significant difference in lung tissues between the TCZ-4 and DEX-10 treatments. There is a need for large-scale studies in which combined drugs (TCZ and DEX) are used in the treatment of ARDS and in which related mechanisms such as inflammation, apoptosis, and necroptosis are investigated.

## Conclusions

OA was found to facilitate a good model of lung damage with its histopathological effects and cytokine storms in lung tissues. In this model of ALI caused by OA, TCZ and DEX appeared to be effective in preventing cytokine storms through the downregulation of the expression of proinflammatory cytokines such as TNF-α, IL-1β, IL-6, and IL-8. OA reduces antioxidant parameters such as SOD, GSH, and CAT in lung tissues, while TCZ and DEX exert protective effects against cell damage by increasing the levels of those antioxidants. We think that studies should be conducted to determine the effects of the use of tocilizumab and dexamethasone on cell death pathways in lung damage caused by oleic acid.

## Data Availability

The datasets used and/or analysed during the current study are available from the corresponding author on reasonable request.

## Abbreviations

OA: Oleic acid
ARDS: Acute respiratory distress syndrome
ALI: Acute lung injury
CAT: Catalase
COVID-19: Coronavirus disease 2019
DAB: 3,3′-Diaminobenzidine tetrahydrochloride
DEX: Dexamethasone
GR: Glutathione reductase
GPx: Glutathione peroxidase
GSH: Glutathione
GSSG: Oxidized glutathione
H_2_O_2_: Hydrogen peroxide
H&E: Hematoxylin and eosin
IL: Interleukin
IL-1β: Interleukin 1 beta
LPO: Lipid peroxidation
MDA: Malondialdehyde
ROS: Reactive oxygen species
SEM: Scanning electron microscopy
SOD: Superoxide dismutase
TCZ: Tocilizumab
TNF-α: Tumor necrosis factor α
